# Design and construction of an electrochemical sensor for the determination of cerium(iii) ions in petroleum water samples based on a Schiff base-carbon nanotube as an ionophore

**DOI:** 10.1039/d1ra08337f

**Published:** 2021-12-21

**Authors:** Tamer Awad Ali, Gehad G. Mohamed

**Affiliations:** Egyptian Petroleum Research Institute (EPRI) 11727 Cairo Egypt dr_tamerawad@yahoo.com +20 10 06890640; Chemistry Department, Faculty of Science, Cairo University 12613 Giza Egypt

## Abstract

A carbon paste sensor (CPE) and screen-printed sensor (SPE) for Ce(iii)-selective determination were prepared using a 2,6-pyridine dicarbomethine-triethylene tetraamine macrocyclic Schiff base ligand (PDCTETA) and multi-walled carbon nanotubes (MWCNTs) as good sensing materials. With respect to most common cations, such as alkali, alkaline earth, transition, and heavy metal ions, the electrodes display high selectivity for the Ce(iii) ion. The sensors respond to Ce(iii) ions in a linear range of 1 × 10^−7^ to 1 × 10^−1^ and 1 × 10^−8^ to 1 × 10^−1^ mol L^−1^ with a slope of 18.96 ± 0.73 and 19.63 ± 0.51 mV per decade change in concentration with a detection limit of 1.10 × 10^−8^ and 5.24 × 10^−9^ mol L^−1^ for CPE (sensor IV) and SPE (sensor VIII), respectively. The sensors were found to have a lifetime of 102 and 200 days. The suggested electrodes performed well throughout the pH ranges of 3.5–8.0 and 3.0–8.5, with response times of 8 and 6 seconds for sensor IV and sensor VIII, respectively. The sensors have been used to measure Ce(iii) ions in water samples from several petroleum wells. They have also been utilized as indicator electrodes in Ce(iii) ion potentiometric titrations with EDTA. The results were quite similar to those obtained by employing atomic absorption spectrometry (AAS).

## Abstract 2

A carbon paste and screen-printed sensor for Ce(iii)-selective determination were prepared using a 2,6-pyridine dicarbomethine-triethylene tetraamine macrocyclic Schiff base ligand and multi-walled carbon nanotubes as good sensing materials.

## Graphical abstract

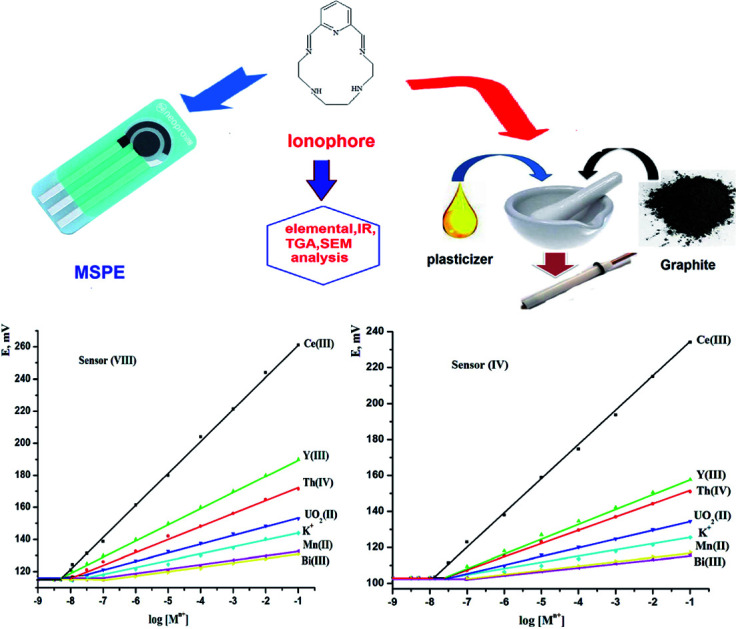

## 1. Introduction

Cerium is the most prevalent element in the lanthanum family of elements.^1^ Cerium is utilized in nuclear reactors, nickel and chromium alloys, microwave devices, lasers, masers, and television sets, among other applications.^2,3^ Cerium is also utilized in agriculture, forestry, and animal husbandry, and the study of cerium in the environment is receiving a lot of interest these days. Humans exposed to cerium inhalation have reported heightened sensitivity to heat, itching, and a greater sense of odour and taste.^4^ The growing use of cerium in industry, as well as reports of cerium toxicity, necessitate the development of analytical techniques for monitoring cerium in the environment.^4^

Many analytical techniques, such as neutron activation analysis,^5^ inductively coupled plasma atomic emission spectrometry (ICP-AES),^6^ and even traditional spectroscopic and fluorimetric methods,^7^ are strong instruments for the measurement of cerium. There are also a number of electrochemical techniques.^8,9^ For most analytical facilities, these procedures are either time intensive, necessitating several analyses, or prohibitively costly. Also, these published methods are known to utilize risky solvents, poor sensitivity, not portable, extensive labor resources, invest long examination energy, necessitate convoluted procedure for the preparation of the sample and sometimes it is important to make sample pretreatments.^10^ Potentiometric sensors are a low-cost, easy-to-use technique for analysing hazardous and heavy metal ions in solution, with good selectivity and sensitivity.^11–14^ Multiwalled carbon nanotubes (MWCNTs) are allotropes of carbon with a cylindrical nanostructure which have fabulous properties. They are used for nanotechnology, electronics, and other fields of materials science due to its high electrical conductivity, thermal conductivity and surface area.^10^ They have been widely used as electrochemical modified sensors for determination of a wide variety of substrates.^10^ Because of their fast speed, cheap cost, easy fabrication, wide dynamic range, and no need of sample pretreatment, ion-selective electrodes (ISE) are ideal devices for detecting the various cationic and anionic species.^15–18^ In addition to utilizing ISEs to determine the studied species directly, they may also be utilized to determine other species indirectly.^16^ Metal ions-sensitive electrodes have been the subject of a lot of research in the last decade.^19^

Carbon paste electrodes (CPEs) have gotten a lot of attention as ion selective electrodes because of their benefits over membrane electrodes including renewability, stability, low ohmic resistance, and no requirement for an internal solution.^20–22^ The majority of CPE-based potentiometric sensors developed thus far are based on the inclusion of a selective chemical into the carbon paste. Graphite powder is mixed in a non-conductive mineral oil to make the carbon paste. Mineral oil incorporation has certain drawbacks for CPEs. Mineral oil isn't component-fixed since its use in a variety of petroleum refining and crude oil processing processes, and certain unaccounted components can have unanticipated effects on detection and analysis.^23–25^ They also have a mechanical issue, with mechanical stability that falls between membrane electrodes and solid electrodes.

The screen-printing process appears to be one of the most promising ways for producing simple, quick, and low-cost biosensors.^26,27^ Biomolecules, insecticides, antigens, and anions have all been detected using biosensors based on screen-printed electrodes.^28^

Because all of the equipment required for electrochemical analysis is portable, electrochemical biosensors based on screen-printed electrodes are in line with the needs of *in situ* screening devices. They feature all of the key biosensor performance qualities, such as minimal sample preparation, apparatus simplicity, and quick findings, and they are also cost effective, compact, and getting downsized with new technologies.^29,30^

The current work explains how to make simple potentiometric sensors and how to use them to measure Ce(iii) in water samples from different petroleum wells. Apart from studying the effect of adding 2,6-pyridine dicarbomethine-triethylene tetraamine macrocyclic Schiff base ligand (PDCTETA) as an ionophore and multi-wall carbon nanotubes (MWCNTs) to graphite for constructing carbon nanotubes modified carbon paste sensors and screen-printed sensors, all of the prepared electrodes were optimised to select the electrode with the most favourable analogue properties. The developed sensors were then used in the potentiometric measurement of Ce(iii) in analytical grade solutions and various petroleum well water samples utilizing standard additions and direct potentiometric methods.

## 2. Experimental

### 2.1. Analysis techniques

The CHNS-932 (LECO) Vario Elemental Analyzer was used to perform carbon, hydrogen, and nitrogen microanalyses at the Microanalytical Center, University of Cairo, Egypt. The molar conductance of the solid complex's 10^−3^ M solution was calculated using a Jenway 4010 conductivity metre. On a PerkinElmer 1650 spectrometer, the FT-IR spectra were captured as KBr pellets (4000–400 cm^−1^). UV-vis spectra were taken using a Shimadzu UVmini-1240 UV-Vis spectrophotometer. The electronic spectra of the compound were obtained at room temperature using a Shimadzu 3101pc spectrophotometer. Thermogravimetric studies of the solid complex (TG and DTG) were all carried out at a rate of 10 °C min^−1^ from ambient temperature to 1000 °C. In the nitrogen atmosphere, a Shimadzu TG-50H thermal analyzer was used. SEM Model Quanta 250 FEG (Field Emission Gun) linked to the EDX Unit (Energy Dispersive X-ray Analysis), 30 kV accelerating voltage, 14× magnification up to 1 000 000, and Gun. In resolution, FEI Agency, Netherlands. The Jenway 3505 pH metre was used to make possible laboratory readings. Different ion selective electrodes were conjugated with the double-junction reference electrode silver–silver chloride (Metrohm 6.0726.100). pH measurements were taken with a Thermo-Orion model Orion 3 stars from the United States. Prior to usage, all of the glassware was thoroughly cleaned with distilled water and dried in the oven.

### 2.2. Reagents

All working solutions were made using double distilled water, and the suggested procedure was utilized to optimize them wherever possible. The macrocyclic Schiff base ligand was synthesized as previously described.^31^ Several electrodes were made with Ce(iii) nitrate (Sigma-Aldrich), carbon graphite powder (synthetic 1–2 m) (Sigma-Aldrich), paraffin oil (Merck), and polyvinyl chloride (PVC) (Aldrich). Magnesium, zinc, calcium, copper, barium, mercury, bismuth, ferric, aluminium, chromium, lanthanum, Yttrium, potassium and sodium chloride salts, as well as anions such as cyanide, bromide, chloride and iodide anions, were used as intermediate materials and were imported from Aldrich.

#### 2.2.1. Samples

Water samples included formation water (samples 1, 2 and 3 were supplied from Badr 1, Western Desert, Badr Petroleum Company (Faras and Raml), Western Desert, Agiba Petroleum Company and Karama, Al-Wahhat-Al-Bahhriyah, Qarun Petroleum Company, respectively) and sample 4, Sidpec Petrochemical Company, Amryia, Alexandria, Egypt.

### 2.3. Screen-printing fabrication

The electrodes were manufactured using a manually operated screen printer and arrays of six pairs of working electrodes (each 5 × 35 mm) on a 0.2 mm polyvinyl chloride flexible sheet.^32,33^ To produce different compositions, the working electrodes were printed using handmade printing carbon ink with PDCTETA as ionophore, multi-wall carbon nanotubes (MWCNTs), paraffin oil, carbon powder and 130 mg of polyvinyl chloride 8%, then kept at 50 °C for 30 minutes and allowed to cool. After that, an insulator layer was placed to the printed electrodes, leaving a rectangular-shaped (5 mm) working area and a comparable region on the opposite side (for electrical contact). The entire sensor production process, which includes three printing and curing cycles for consecutive films, takes less than an hour. The electrodes were made, kept at 4 °C, and immediately utilized in potentiometric testing.

### 2.4. Preparation of carbon paste modified sensors

Bare CPEs were constructed by mixing of different amount of graphite powder with paraffin oil in a mortar and pestle heated at 700 °C in the muffle furnace for 10 seconds. In a modified paste prepared in the same way, the graphite powder was mixed to achieve a new composition with the required quantity of PDCTETA as ionophore and multi-wall carbon nanotubes (MWCNTs). The two modified pastes were placed inside a 2.5 mm diameter polyethylene tube with a cut-off tip. A thorough flank of copper wire was inserted into the paste to establish electrical contact. The freshly modified carbon paste sensors surface was preconditioned for 1 hour before being washed with deionized water and exposed to a 1.0 × 10^−3^ mol L^−1^ Ce(iii) ion solution. By pushing more out, a new electrode surface was obtained. With a glass rod, the excess paste was removed, and the exposed end was polished on a paper until it was glossy.^34,35^

### 2.5. Potentiometric electrodes and emf measurements

All the emf observations were made relative to Ag/AgCl electrode with a pH/mV meter. The emf measurements were carried out with the following cell assembly:

Ag/AgCl|sample solution|CPE or SPE.

The performance of the electrodes was investigated by measuring the emfs of cerium nitrate solution which is prepared with a concentration range of 1.0 × 10^−8^ to 1.0 × 10^−1^ mol L^−1^ by serial dilution. Each solution was stirred and the potential was recorded when it became stable, and then plotted as a logarithmic function of Ce(iii) activity.

#### 2.5.1. Response time

When the electrode was submerged in the solution to be examined, the potential was measured as a function of time to determine its response time. The electrode was initially immersed in a 1 × 10^−8^ mol L^−1^ cerium nitrate solution (pH = 5) before being switched to a 1 × 10^−7^ mol L^−1^ solution of the same ion (pH = 5). The potential of the solution was measured at intervals of 7 seconds after dipping the electrode in the second solution, which was referred to as zero time. The potentials were plotted against time to determine the electrode's reaction time (seconds).

#### 2.5.2. Effect of pH

A series of solutions with different pH (1–10) were produced to examine the influence of pH on the potential of a given concentration of solution while maintaining the Ce(iii) ion concentration constant (1 × 10^−3^ and 1 × 10^−5^ mol L^−1^). Dilute HCl or dilute NaOH solutions were used to alter the pH of the solution. For each pH value, the potential of solution was measured and plotted against the pH.

#### 2.5.3. Effect of temperature

Temperature had an effect on the performance of the modified CPEs and SPEs, which were tested in a thermostat at temperatures ranging from 10 to 60 °C.

#### 2.5.4. Life span of the paste

The electrode responses were recorded over one or more weeks over time and a response curve was created using the data in this investigation. The electrode response curve revealed that the response does not change over time. After this time, the electrode begins to behave abnormally and cannot be utilized for any measurements. The life of an electrode is the duration during which the electrode response remains consistent.

## 3. Results and discussion

### 3.1. Characterization of macrocyclic Schiff base

**Scheme 1:**
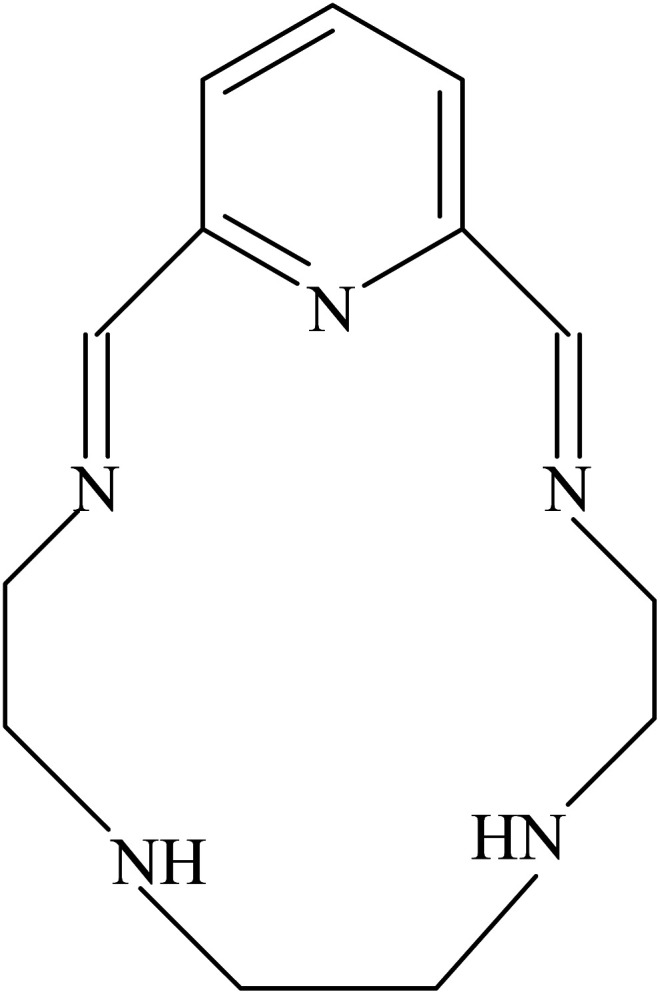
Structure of the macrocyclic Schiff base ionophore.

The results of elemental analyses (C, H and N) with molecular formula and the melting point are presented as described previously.^31^ The results obtained are in good agreement with those calculated for the suggested formula and the melting point is sharp indicating the purity of the prepared Schiff base. The structure of this Schiff base (Scheme 1) is also confirmed by IR, mass and ^1^H NMR spectra, which were discussed in detailed manner.^31^

### 3.2. Preliminary study

**Fig. 1:**
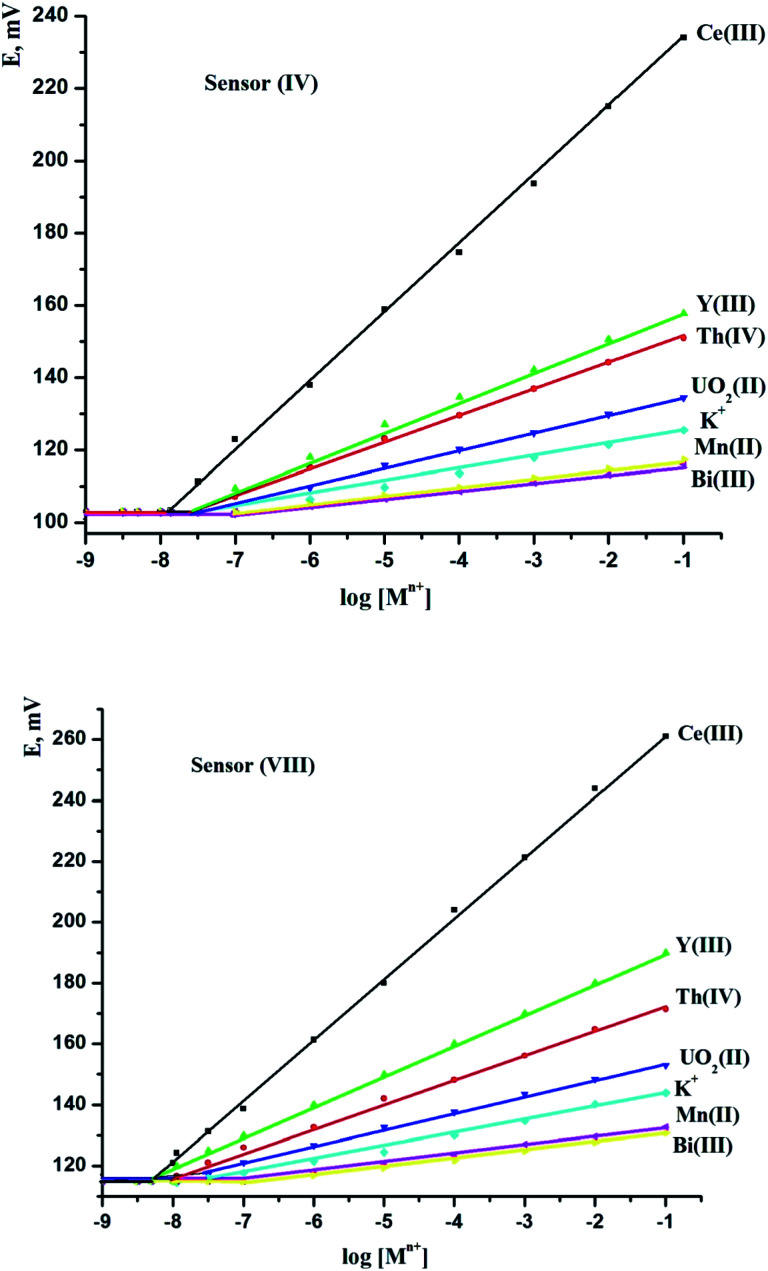
Potential responses of various cation-modified carbone paste sensor (sensor IV) and screen-printed electrodes (sensor VIII) based on the prepared 2,6-pyridine dicarbomethine-triethylene tetraamine macrocyclic Schiff base ligand (PDCTETA) as ionophore.

It was employed as a neutral carrier to build CPS and SPS sensors for a broad variety of cations, including alkali, alkaline earth, transitional, and heavy metal ions, to examine the potential response and selectivity of the PDCTETA ionophore for different metal ions. Fig. 1 shows the potential responses of different ion selective electrodes depending on PDCTETA ionophore. Except for the Ce(iii) ion selective electrode, the slope of the related potential–pM graphs was significantly lower than the predicted Nernstian slopes of 59.6, 29.8, and 19.8. mV per decade for mono-, di-, and tri-valent cations, respectively, despite the concentration range being relatively limited. The Ce(iii) ion with the most sensitive response across a wide concentration range appears to be adequately determined with the CPS and SPS sensors based on ionophore, as shown by the obtained findings. This is most likely owing to the PDCTETA inophore has strong selectivity for Ce(iii) over other metal ions.

### 3.3. Composition and characteristics of the sensors

**Fig. 2:**
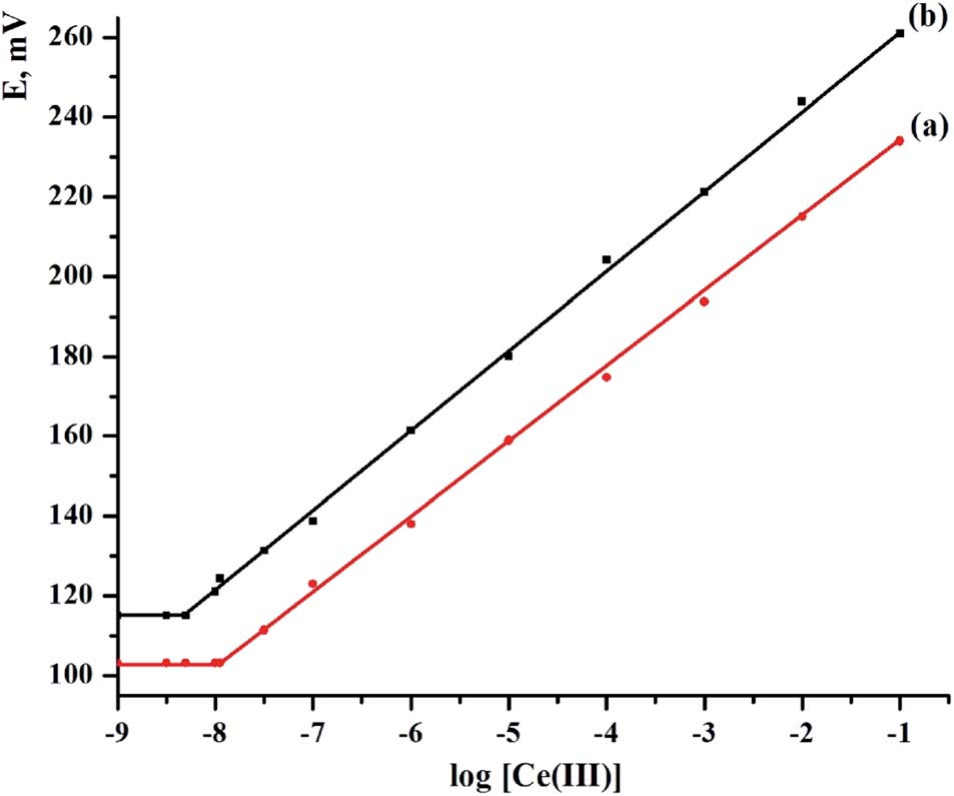
Calibration curve for (a) CPS (sensor IV) and (b) SPS (sensor VIII) at *T* = 25 °C, pH = 5.

The electrode response for Ce(iii) ions is well known to be dependent on the number and type of the electrode components.^23,25,27^ The sensitivity, detection limit, linear range, and selectivity coefficients of a sensor are used to define its general properties. The kind and amount of ionophore, as well as the amount of paraffin oil, are claimed to be key characteristics of modified carbon paste and screen-printed sensors. In order to identify the optimum electrode composition, five CPEs and five SPEs sensors were made. The weights of PDCTETA were varied as 5.0, 7.5, 10, 12 and 15 mg (w/w%). The resulting slopes are found to be 10.55 ± 1.47, 15.27 ± 1.12, 17.88 ± 0.92, 18.96 ± 0.73 and 17.41 ± 0.89 mV per decade for CPEs from sensors I to V, respectively, and 12.39 ± 1.05, 18.72 ± 0.64, 19.63 ± 0.51, 18.21 ± 0.73, and 16.40 ± 1.03 mV per decade for SPEs from sensors VI to X, respectively. Table 1 shows that the linear range was 1.0 × 10^−7^ to 1.0 × 10^−1^ and 1.0 × 10^−8^ to 1.0 × 10^−1^ mol L^−1^ for sensors IV and VIII, respectively. These findings demonstrate that for sensors IV and VIII, the modified electrodes containing 12.0 and 10.0 mg ionophore had a greater Nernstian slope and a wider range of linearity. This proportion was determined as the best for the production of cerium electrodes. Experiments were repeated numerous times to ensure that the results were consistent. For five sets of tests, EMFs were plotted against the log of cerium ion activity, and calibration curves were constructed, yielding a standard variation of ±0.52–1.74 mV. According to IUPAC guidelines,^36^ the detection limit of the sensors was estimated by intersecting two extrapolated linear sections of the curve^23,25,27^ and was found to be 1.10 × 10^−8^ and 5.24 × 10^−9^ mol L^−1^ for sensor (IV) and sensor (VIII), respectively (Fig. 2 and Table 2).

**Table 1:** Optimization of the carbon paste (CPE) and screen-printed sensors (SPE) ingredients

Composition% (w/w)					Electrode characteristics		
No.	GP^a^	PO^b^	(I)^c^	MWCNTs^d^	Slope (mV per decade)	LR (mol L^−1^)	*R*
**CPE sensor**							
I	61	34	5	—	10.55 ± 1.47	1.0 × 10^−5^ to 1.0 × 10^−1^	0.911
II	59	33.5	7.5	6	15.27 ± 1.12	1.0 × 10^−6^ to 1.0 × 10^−1^	0.929
III	58	32	10	7	17.88 ± 0.92	2.7 × 10^−7^ to 1.0 × 10^−1^	0.988
**IV**	**57**	**31**	**12**	**8.5**	**18.96 ± 0.73**	**1.0 × 10** ^**−7**^ **to 1.0 × 10** ^**−1**^	**0.997**
V	52	33	15	10	17.41 ± 0.89	1.0 × 10^−7^ to 1.0 × 10^−1^	0.992

**SPE sensor**							
VI	58	37	5	—	12.39 ± 1.05	1.0 × 10^−6^ to 1.0 × 10^−1^	0.976
VII	57	35.5	7.5	5	18.72 ± 0.64	5.0 × 10^−8^ to 1.0 × 10^−1^	0.994
**VIII**	**58**	**32**	**10**	**6.5**	**19.63±0.51**	**1.0 × 10** ^**−8**^ **to 1.0 × 10** ^**−1**^	**0.999**
IX	57	31	12	7.5	18.21 ± 0.73	1.0 × 10^−8^ to 1.0 × 10^−1^	0.991
X	53	32	15	9	16.40 ± 1.03	5.0 × 10^−8^ to 1.0 × 10^−1^	0.980

a Graphite powder.

b Paraffin oil.

c 2,6-Pyridine dicarbomethine-triethylene tetraamine macrocyclic Schiff base ligand (PDCTETA).

d Multi-walled carbon nanotubes (MWCNTs).

**Table 2:** Response characteristics of CPE (sensor IV) and SPE (sensor VIII) potentiometric sensors

Parameter	CPE SPE	
	Sensor IV	Sensor VIII
Slope (mV per decade)	18.96 ± 0.73	19.63 ± 0.51
Correlation coefficient, *r*	0.997	0.999
Lower detection limit (mol L^−1^)	1.10 × 10^−8^	5.24 × 10^−9^
Response time (s)	8	6
Working pH range	3.5–8.0	3.0–8.5
Usable range (mol L^−1^)	1 × 10^−7^ to 1.0 × 10^−1^	1 × 10^−8^ to 1 × 10^−1^
Isothermal coefficient (V °C^−1^)	0.000107	0.000168
Intercept (mV)	251.35 ± 1.18	283.46 ± 1.21
Life time (days)	102	200
Accuracy (%)	99.12	99.97
Precision (%)	0.196	0.082

### 3.4. Surface characterization

Surface interaction between the selected macrocyclic Schiff base ligand (PDCTETA ionophore) and the Ce(iii) ions is important for selective extraction of the target ion from the sample solution into the paste during potential measurements.

**Fig. 3:**
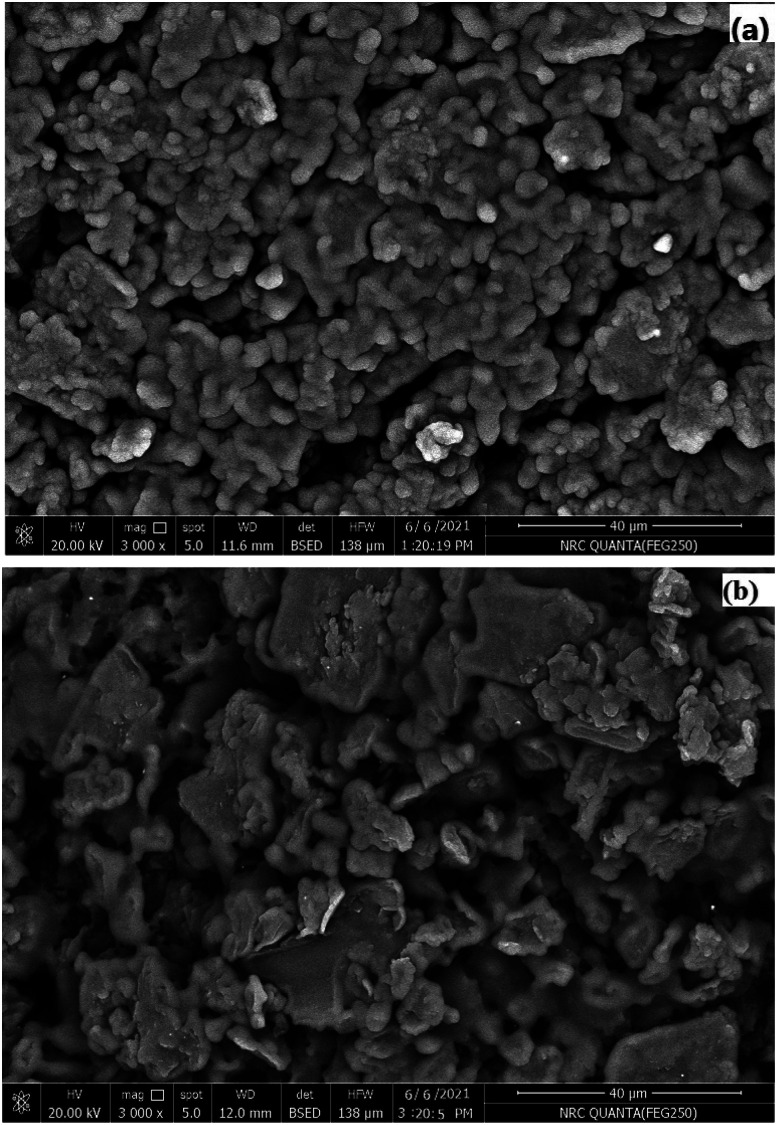
SEM images for PDCTETA-SPE surface (a) before and (b) after soaking in 1.0 × 10^−3^ mol L^−1^ Ce(iii) ions solution.

As a consequence, a scanning electron microscope was used to characterize the surface morphology of the suggested SPE sensor (VIII), which is a suitable instrument for evaluating the surface morphology of an ion-selective electrode. Fig. 3 shows SEM images of the manufactured electrode before and after soaking in 1.0 × 10^−3^ mol L^−1^ Ce(iii) ions solution (a and b).

After soaking the suggested SPE in Ce(iii) ions solution, less gaps between surface components were detected as a result of the interaction between the Ce(iii) ions and the active sites groups of the macrocyclic ligand (PDCTETA) that occupied the space between the surface components (Fig. 3b).

### 3.5. Response time of the electrodes

**Fig. 4:**
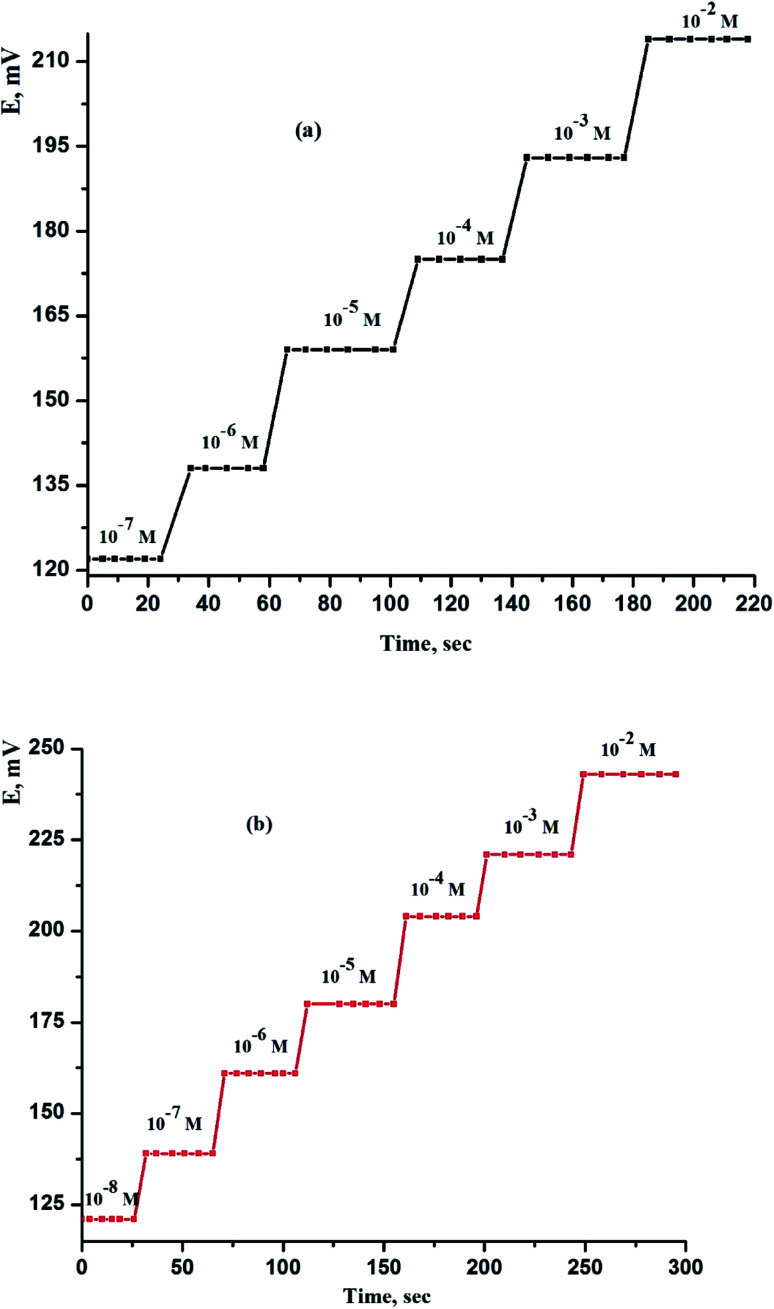
Dynamic response of (a) CPS (sensor IV) and (b) SPS (sensor VIII) obtained by successive increase of Ce(iii) ion concentration at *T* = 25 °C and pH = 5.

For every analytical application, the reaction time of CPEs and SPEs sensors is particularly critical. After consecutive immersions in a series of solutions, each with a 10-fold concentration variation, the average reaction time was determined as the time it took for the electrodes to achieve a cell potential of 90% of the final equilibrium values. The practical reaction time was measured in this work by varying the Ce(iii) ion concentration in solution throughout a range of concentrations from 1.0 × 10^−7^ to 1.0 × 10^−2^ and 1.0 × 10^−8^ to 1.0 × 10^−2^ mol L^−1^ for sensor (IV) and sensor (VIII), respectively. Fig. 4 depicts the real potential *versus* time traces. As can be observed, the sensors finds its equilibrium response in a very short period, which is determined to be 8 and 6 s for sensor (IV) and sensor (VIII), respectively, throughout the whole concentration range. The fact that these electrodes include CPEs and SPEs sensors enclosed by a very thin coating of paraffin oil and serving as a conductor, as well as the absence of an internal reference solution, explains the rapid reaction times.

### 3.6. Effect of pH

**Fig. 5:**
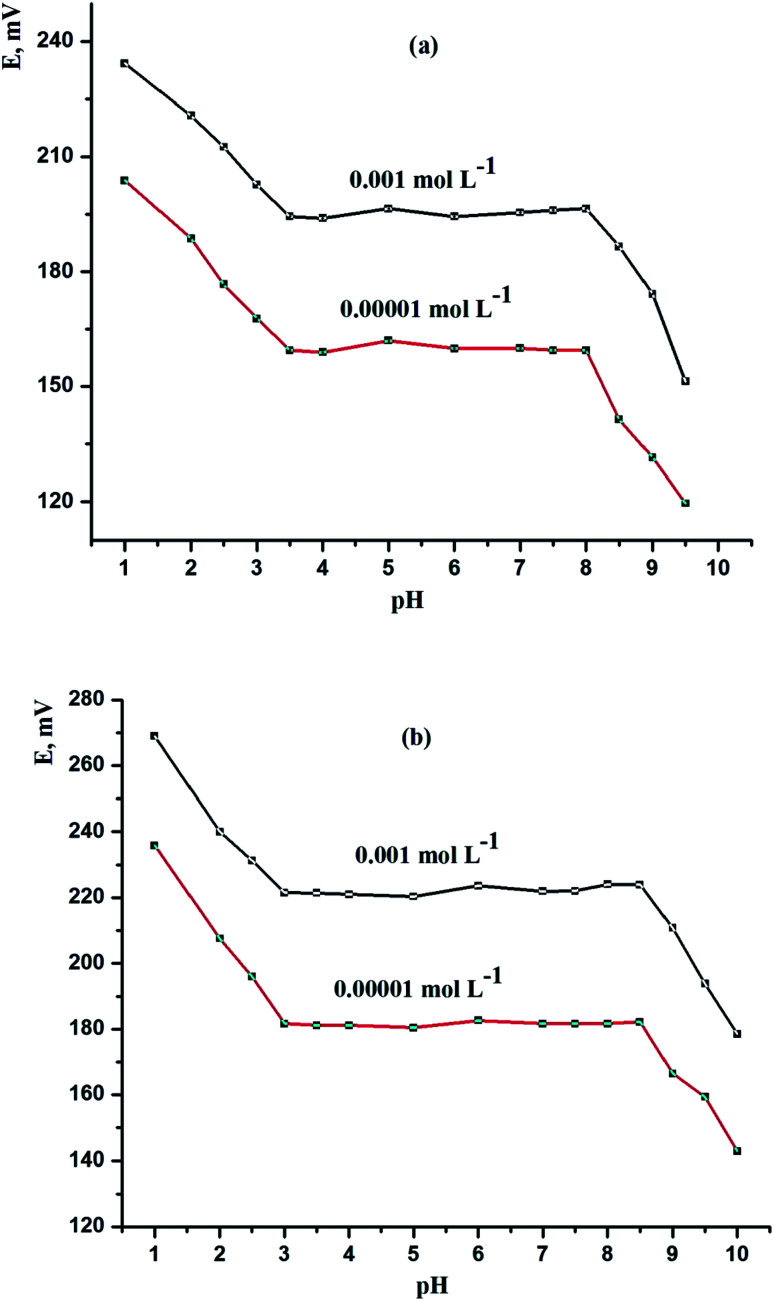
Effect of pH of the test solution on the performance characteristics of (a) CPS (sensor IV) and (b) SPS (sensor VIII) at *T* = 25 °C and [Ce(iii)] = 1 × 10^−3^ and 1 × 10^−5^ mol L^−1^.

In the pH range of 1.0 to 10.0, the influence of pH on potential was investigated. A series of solutions with varying pH were produced for this, with the concentration of Ce(iii) being constant (1.0 × 10^−3^ and 1.0 × 10^−5^ mol L^−1^). 0.01 mol L^−1^ HNO_3_ or sodium hydroxide solutions were used to alter the pH of the solutions. The findings are shown in Fig. 5. In the pH ranges of 3.5–8.0 and 3.0–8.5, respectively, as the operating pH ranges for sensor (IV) and sensor (VIII), the potential stays constant, as shown in Fig. 5. The production of cerium hydroxide causes precipitation above pH 8.5. At pH levels less than 3.0, on the other hand, the electrode responds more to hydrogen ions than to Ce(iii) in solution. This is most likely owing to the electrodes' simultaneous reaction to H_3_O^+^ and Ce(iii) ions.

### 3.7. Influence of test solution temperature

**Fig. 6:**
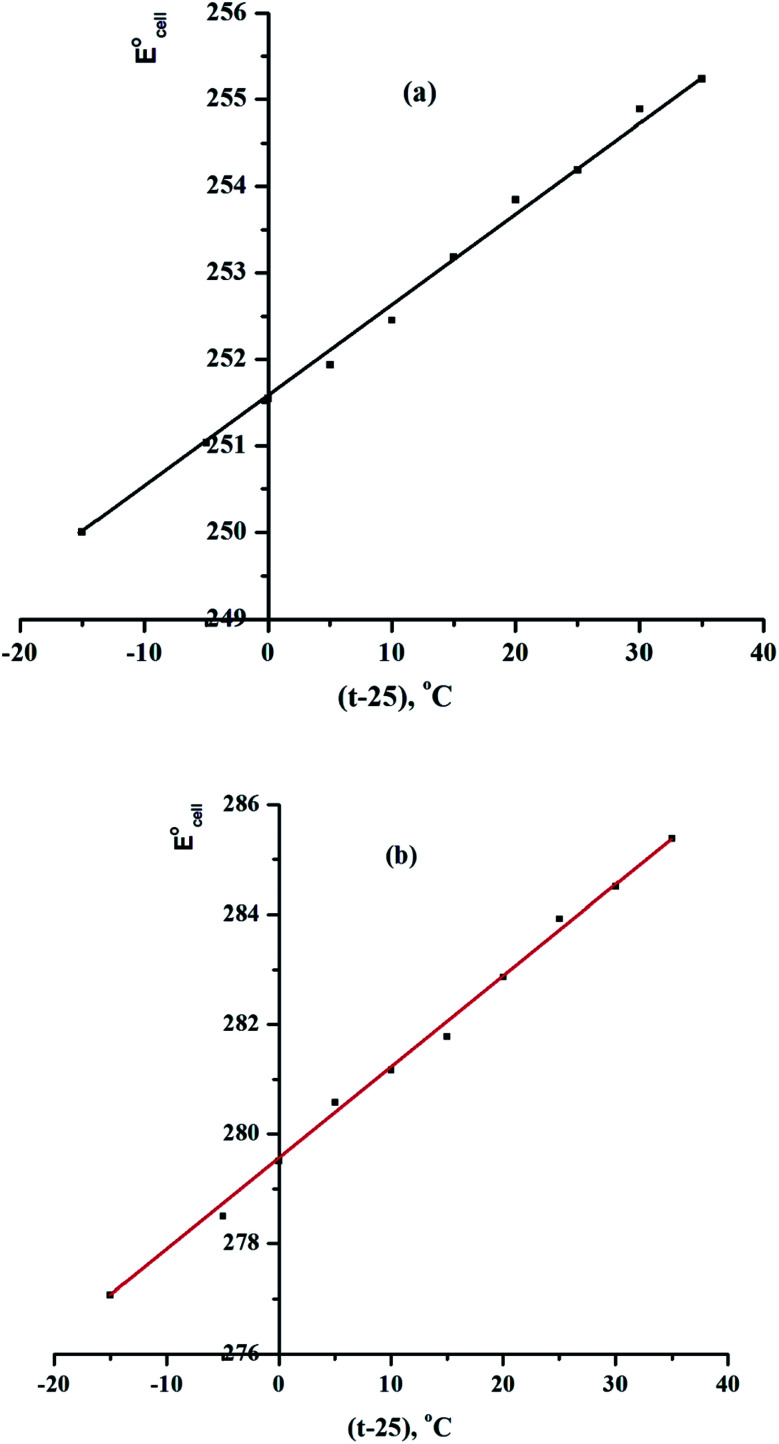
Effect of temperature on the performance of (a) CPS (sensor IV) and (b) SPS (sensor VIII) at pH = 5.

The improved Ce-ISE was tested in test solutions at various temperatures (10, 20, 30, 40, 50, and 60 °C) in this investigation. In the temperature range of 10 to 60 °C, the electrodes displayed excellent Nernstian behaviour. The standard cell potentials 
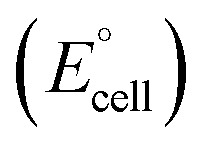
 were calculated from the calibration plots at various temperatures as the intercepts of these plots at *p*Ce(iii) = 0 and used to calculate the cell's isothermal temperature coefficient (d*E*°/d*t*) using the equation:
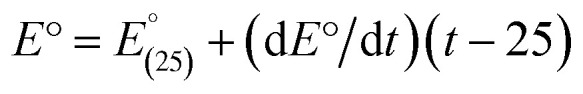
where 
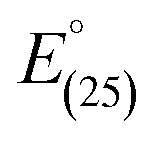
 is the standard electrode potential at 25 °C, and the slope of the straight-line produced reflects the electrodes' isothermal coefficient, which is determined to be 0.000107 and 0.000168 V °C^−1^, respectively, for sensors IV and VIII (Fig. 6).^37–39^ The obtained isothermal coefficients of the electrodes suggested that they had relatively good thermal stability within the examined temperature range and did not deviate from predicted Nernstian behaviour.

### 3.8. Effect of life span of the paste

**Fig. 7:**
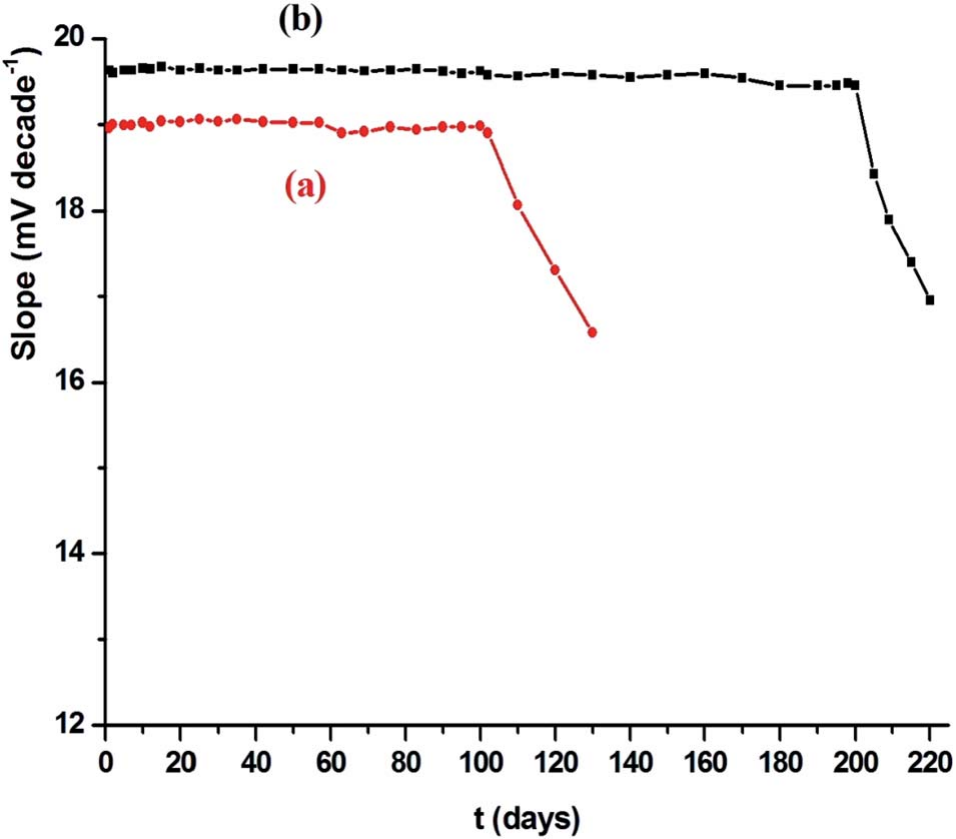
Life time of the Ce(iii) modified of (a) CPS (sensor IV) and (b) SPS (sensor VIII) at *T* = 25 °C and pH = 5.

The calibration diagram was developed under ideal conditions over a long period of time to estimate the life duration of the modified CPE (sensor IV) and SPS (sensor VIII) sensors. The proposed improved IV and VIII sensors had a life duration of 102 and 200 days, respectively, with no significant change in the calibration diagram slopes, as shown in Fig. 7. After this time, the calibration diagrams slope dropped considerably. To confirm the repeatability of the manufactured Ce(iii)-CPE and Ce(iii)-SPE sensors, the best content of the sensing electrode material was produced, and their performances were evaluated in interaction with Ce(iii) ions. The results presented the average of slopes and detection limits as from 18.96 ± 0.73 to 16.58 ± 0.96 and 19.63 ± 0.51 to 16.96 ± 0.57 mV per decade and 1.10 × 10^−8^ to 2.51 × 10^−7^ and 5.24 × 10^−9^ to 9.85 × 10^−8^ mol L^−1^ for sensors IV and VIII, respectively.

### 3.9. Selectivity

The proportional reactivity of an electrode to the main ion over other ions in the solution determines its potentiometric selectivity coefficient. The matched potential method (MPM) and fixed interference technique (FIM) (approved by IUPAC) were used to calculate the potentiometric selectivity coefficients.^40^ The activity of Ce(iii) was *a*_A_ = 5.0 × 10^−6^ and 
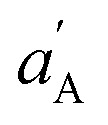
 = 1.0 × 10^−3^ mol L^−1^, respectively, according to the MPM, and the associated variations in potential (*E*/mV) were measured. Following that, a solution of an activity *a*_B_ interfering ion was added to the reference cerium solution until the same potential change (*E*/mV) was seen. The selectivity coefficient (*K*^MPM^_A,B_), for interferences was calculated using the following equation:
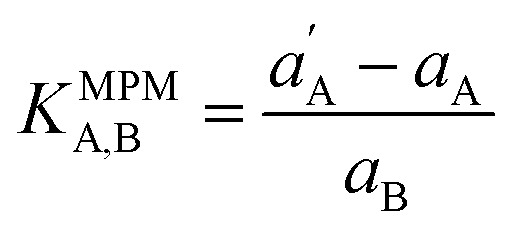


The fixed interference technique was being used to measure the selectivity coefficients of interfering species (FIM). The modified-CPS and SPS sensors, as well as the reference electrode, were immersed in 25.0 mL of 1.0 × 10^−3^ mol L^−1^ interference ion solution in this manner. Varied amounts of Ce(iii) solution ranging from 1.0 × 10^−5^ to 1.0 × 10^−3^ mol L^−1^ were added by micro syringe to achieve various cerium ion concentrations ranging from 1.0 × 10^−8^ mol L^−1^ to 1.0 × 10^−2^ mol L^−1^. The solution was magnetically swirled throughout, and the cell potential was measured after each addition. The concentration of the interference ion (M^*n*+^) was maintained in this manner without the need to change the electrode to new solutions. The pH of all solutions was constant at about 5.0, and then, the fixed interference method selectivity coefficient was given by:
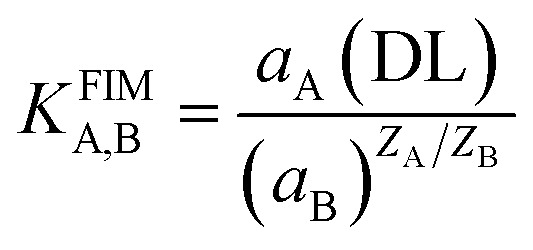
where *a*_A_ (DL) is the primary ion activity in the detection limit, *a*_B_ is the interference ion activity (1.0 × 10^−3^ mol L^−1^), *Z*_A_ and *Z*_B_ are the charges of the primary and interference ions, respectively. Table 3 summarizes all of the findings. The selectivity coefficients of the electrodes are in the order of 1.1 × 10^−2^ or smaller for all ions, indicating that they would not significantly disrupt the Ce(iii) selective membrane sensor's performance. The selectivity coefficient is known to be dependent on the ionophore's ion binding capabilities as well as the composition of modified-CPE and SPE sensors. These low values of selectivity coefficient may be also attributed to the fact that increasing the ionic strength will lower the ionic activity coefficient and hence the activity of primary ion (Ce(iii) ion). This decrease in the activity of the primary ion alone produces a decrease in potential which counteracts to some extent the increase in potential due to the interfering ions.

**Table 3:** Selectivity coefficients of various ions using sensor (IV) and sensor (VIII)

Interfering ions	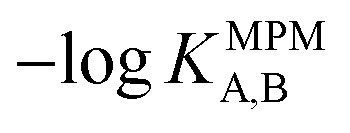		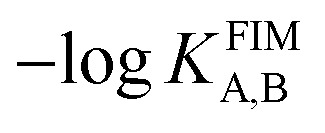	
	Sensor (IV)^a^	Sensor (VIII)^a^	Sensor (IV)^b^	Sensor (VIII)^b^
Mg^2+^	5.06	5.15	5.22	5.27
Zn^2+^	5.13	5.19	5.26	5.41
Ca^2+^	5.54	5.61	5.59	5.62
Cu^2+^	4.98	5.15	5.21	5.36
Ba^2+^	4.57	5.59	5.65	5.68
Hg^2+^	2.33	2.61	3.19	3.26
Bi^3+^	3.91	3.94	3.98	4.04
Fe^3+^	2.13	2.25	2.30	2.42
Al^3+^	3.59	3.62	3.65	3.68
Cr^3+^	5.32	5.37	5.39	5.41
K^+^	5.87	5.89	5.90	5.92
Na^+^	5.96	6.11	6.02	6.27
Y^3+^	2.21	2.23	2.35	2.38
La^3+^	2.65	2.68	2.71	2.73
CN^−^	5.77	5.79	5.82	5.84
Br^−^	5.06	5.08	5.11	5.17
Cl^−^	4.84	4.86	4.89	4.93
I^−^	4.93	5.11	5.29	5.33

a Selectivity coefficients found by matched potential method.

b Selectivity coefficients found by fixed interference method.

### 3.10. Analytical applications

The suggested modified-CPE and SPE sensors were successfully used to determine Ce(iii) ion directly in several petroleum water samples. The direct potentiometric calibration and standard addition techniques were used for the analyses. The findings of each water sample analysis revealed that the determination of Ce(iii) ion was accurate enough, and they were compared to those obtained by AAS (Table 4). This demonstrates that the modified-CPE (sensor IV) and SPE (sensor VIII) sensors can be used to determine Ce(iii) ions in a variety of petroleum water samples. The percentage recovery and low standard and relative standard deviation values supported the accuracy and precision of the prosed potentiometric method. The obtained results listed in Table 4 suggested that there is no significant difference between the proposed and AAS method as pointed out from the values of *F*- and *t*-test.

**Table 4:** Determination of Ce(iii) ions in spiked water samples using sensor (IV) and sensor (VIII)

	[Ce(iii)] (mg L^−1^)									
	CPS (sensor IV)				SPS (sensor VIII)			AAS		
	Added	Found	R.S.D (%)	Recovery (%)	Found	R.S.D (%)	Recovery (%)	Found	R.S.D (%)	Recovery (%)
**(a) Standard addition method** a										
1	0.45	0.442	1.078	98.22	0.446	0.571	99.11	0.44	1.162	97.78
	0.60	0.591	1.013	98.50	0.595	0.544	99.17	0.589	1.096	98.17
2	0.30	0.295	1.017	98.33	0.298	0.378	99.33	0.293	1.107	97.67
	0.35	0.346	1.032	98.85	0.349	0.328	99.71	0.344	1.021	98.29
3	0.25	0.245	1.113	98.0	0.248	0.658	99.2	0.243	1.286	97.2
	0.40	0.393	1.109	98.25	0.397	0.641	99.25	0.391	1.211	97.75
4	0.35	0.345	1.007	98.57	0.351	0.246	100.28	0.343	1.072	98.0
	0.50	0.496	0.993	99.2	0.499	0.341	99.8	0.494	1.005	98.8

**(b) Direct potentiometric method** b										
1	0.45	0.444	0.748	98.67	0.447	0.412	99.33	0.441	1.041	98.0
	0.60	0.594	0.487	99.0	0.598	0.357	99.67	0.592	1.003	98.67
2	0.30	0.297	0.502	99.0	0.299	0.338	99.67	0.294	1.120	98.0
	0.35	0.348	0.419	99.42	0.351	0.210	100.2857	0.347	0.783	99.14
3	0.25	0.247	1.023	98.80	0.249	0.399	99.60	0.244	1.502	97.60
	0.40	0.398	0.384	99.50	0.402	0.197	100.5	0.395	1.077	98.75
4	0.35	0.347	0.438	99.14	0.350	0.263	100.0	0.346	1.123	98.86
	0.50	0.497	0.396	99.40	0.498	0.209	99.60	0.495	0.804	99.0

a *F*-Test = 0.84–1.75 (for sensor IV) and = 0.67–1.48 (for sensor VIII) (tabulated *F* value at 95% confidence limit = 5.91 for *n* = 5). *t*-Test = 1.07–1.88 (for sensor I) and = 0.91–1.63 (for sensor II) (tabulated *t* value at 95% confidence limit = 3.27 for *n* = 5).

b *F*-Test = 0.47–1.32 (for sensor IV) and = 0.26–1.05 (for sensor VIII) (tabulated *F* value at 95% confidence limit = 4.03 for *n* = 5). *t*-Test = 0.55–1.48 (for sensor I) and = 0.31–1.19 (for sensor II) (tabulated *t* value at 95% confidence limit = 1.94 for *n* = 5).

### 3.11. Method validation

#### 3.11.1. Limit of quantification and limit of detection

The limit of quantification (LOQ) was obtained by determining the lowest concentration that can be detected using ICH Q2 (R1) guidelines, below which the calibration range becomes non-linear. For CPS (sensor IV) and SPS (sensor VIII), the linearity was determined to be 1.0 × 10^−7^ to 1.0 × 10^−1^ and 1.0 × 10^−8^ to 1.0 × 10^−1^ mol L^−1^, respectively. The limit of detection (LOD) for CPE (sensor IV) and SPE (sensor VIII) was obtained by assessing the lowest concentration of the Ce(iii) ion analyte that can be readily identified as well as the intersection between the two linear lines, and was found to be 1.10 × 10^−8^ and 5.24 × 10^−9^ mol L^−1^, respectively. The LOQ was computed using the formulae below (ICH 2005):

1 LOQ = 10(SD/*S*)

2 LOD = 3(SD/*S*)

where (SD) is the standard deviation of the intercept of the regression line and (*S*) is the slope of the calibration curve. Knowing the LOD, LOQ can be calculated.

#### 3.11.2. Precision

When the technique is done repeatedly to numerous samplings of a homogenous sample, precision is defined as a measure of how near the findings are to one another. The precision of the replicate analysis is generally reported as standard deviation (SD) or relative standard deviations (RSD percent). For the measurement of Ce(iii) in various water samples, intra-day (on the same day) and inter-day (on separate days) accuracy were reached utilising two concentrations and five replicates (*n* = 5) of each concentration. The low SD and RSD percent values suggest that the proposed technique has adequate repeatability and reproducibility, as shown in Table 5.

**Table 5:** Evaluation of intra- and inter-days precision and accuracy of CPE (sensor IV) and SPE (sensor VIII) in different water samples

Electrode type	Sample no.	[Ce(iii)] taken, (mg L^−1^)	Intra day			Inter day		
			[Ce(iii)] found, (mg mL^−1^)	Recovery (%)	RSD%	[Ce(iii)] found, (mg L^−1^)	Recovery (%)	RSD%
Sensor IV	2	0.30	0.297	99.0	0.574	0.295	98.33	0.743
		0.35	0.348	99.43	0.518	0.344	98.28	0.847
	4	0.35	0.347	99.14	0.498	0.346	98.86	0.698
		0.50	0.495	99.0	0.502	0.494	98.8	0.609
Sensor VIII	2	0.30	0.298	99.33	0.553	0.296	98.67	1.002
		0.35	0.349	99.71	0.499	0.345	98.57	1.063
	4	0.35	0.349	99.71	0.452	0.347	99.14	0.742
		0.50	0.497	99.4	0.511	0.496	99.2	0.704

#### 3.11.3. Robustness/ruggedness of the electrode

The CPS (sensor IV) and SPS (sensor VIII) sensors are resistant to mechanical shocks when heated to 60 °C, and they have the benefit of having their surface renewed without losing their characteristics.

#### 3.11.4. Repeatability/reproducibility

The method's repeatability was tested by monitoring the potential response of varying concentrations of cerium over a 5 day time span. Over the course of five days, the measurement solution's repeatability was determined to be within 0.7 mV.

### 3.12. Comparative study

Table 6 compares the linear range, detection limit, slope, pH range, and response time of previously reported Ce(iii)-selective electrodes^15–19^ to the proposed electrode for comparison purposes. The results in these tables demonstrate that, in many situations, the proposed electrodes' performances are superior to those previously reported electrodes.

**Table 6:** Comparing some of the Ce(iii)-CPE (sensor IV) and Ce(iii)-SPE (sensor VIII) characteristics with some of the previously reported Ce(iii)-ISEs

References	Slope (mV per decade)	Response time (s)	pH	Life time (months)	Linear range (mol L^−1^)	DL (mol L^−1^)
Proposed sensor IV	18.96	8	3.5–8.0	> 1	1.0 × 10^−7^ to 1.0 × 10^−1^	1.10 × 10^−8^
Proposed sensor VIII	19.63	6	3.0–8.5	>6	1.0 × 10^−8^ to 1.0 × 10^−1^	5.24 × 10^−9^
15	19.3	10	5.0–8.0	—	1.0 × 10^−8^ to 1.0 × 10^−1^	2.1 × 10^−9^
16	19.79	5	3.0–7.5	>4	2.0 × 10^−8^ to 1.0 × 10^−1^	6.45 × 10^−9^
17	19.8	25	4.0–8.0	—	1.0 × 10^−5^ to 1.0 × 10^−2^	1.44 × 10^−6^
18	19.6	<10	3.1–9.8	<3	1.0 × 10^−6^ to 1.0 × 10^−1^	5.7 × 10^−7^
18	19.5	12	3.5–7.5	3	1.4 × 10^−7^ to 1.0 × 10^−1^	8.3 × 10^−8^
19	19.7	10	2.5–8.5	5	1.0 × 10^−8^ to 1.0 × 10^−1^	7.7 × 10^−9^

## 4. Conclusions

The suggested Ce(iii)-selective CPE (sensor IV) and SPE (sensor VIII) based on a macrocyclic Schiff base and multi-wall carbon nanotubes (MWCNTs) ionophores might be utilized as a helpful analytical instrument and intriguing alternative for determining Ce(iii) ions in various water samples, according to this research. The sensors demonstrated high sensitivity, a low detection limit, adequate selectivity, long-term stability, and application over a broad pH range.

The sensors (CPE (sensor IV) and SPE (sensor VIII)) have similar linear range, detection limit, lifespan and reaction time, and most importantly, selectivity to other common ion selective electrodes (PVC membrane) holding an internal solution. Another significant benefit of the suggested electrode is that it may be used in both aqueous and partially non-aqueous environments. The electrode allows for direct detection of Ce(iii) ions in a variety of water samples. The values of *F*- and *t*-test confirmed the successful application of the proposed electrodes in Ce(iii) ion determination in petroleum water samples.

## Ethical statement

All experiments were performed in accordance with the ethical rules approved by the ethics committee at the Egyptian Petroleum Research Institute.

## Conflicts of interest

The authors declare that they have no known competing financial interests or personal relationships that could have appeared to influence the work reported in this paper.

## Supplementary Material
